# Rock Sparrow Song Reflects Male Age and Reproductive Success

**DOI:** 10.1371/journal.pone.0043259

**Published:** 2012-08-23

**Authors:** Erwin Nemeth, Bart Kempenaers, Giuliano Matessi, Henrik Brumm

**Affiliations:** 1 Max Planck Institute for Ornithology, Communication and Social Behaviour Group, Seewiesen, Germany; 2 University of Vienna, Department of Behavioural Biology, Vienna, Austria; 3 University of Copenhagen, Department of Biology, Animal Behaviour Group, Copenhagen, Denmark; Pennsylvania State University, United States of America

## Abstract

The evolution of mating signals is closely linked to sexual selection. Acoustic ornaments are often used as secondary sexual traits that signal the quality of the signaller. Here we show that song performance reflects age and reproductive success in the rock sparrow (*Petronia petronia*). In an Alpine population in south-east France, we recorded the songs of males and assessed their genetic breeding success by microsatellite analysis. In addition to temporal and spectral song features, we also analysed for the first time whether the sound pressure level of bird song reflects reproductive success. Males with higher breeding success sang at a lower rate and with a higher maximum frequency. We found also that older males gained more extra-pair young and had a higher overall breeding success, although they also differed almost significantly by having a higher loss of paternity in their own nests. Older males could be distinguished from yearlings by singing at lower rate and higher amplitudes. Our findings suggest that song rate may be used as a signal of age and together with song pitch as a signal of reproductive success in this species. Alternatively, younger and less successful males might try to compensate their inferior status by increased song rates and lower pitch. Independent of age and quality, high-amplitude songs correlated with paternity loss in the own nest, suggesting that in this species song amplitude is not an indicator of male quality but high-intensity songs may be rather a response to unfaithful social mates.

## Introduction

Birdsong is a multifaceted mating signal, and several different acoustic characteristics have been related to its sexual function [1]. For example, song complexity or repertoire size (e.g. [2], [3], but see [4]), special song elements [5], performance-related song variables [6], [7], the timing of singing [8], or combined features [9] have all been found to be an indicator of male quality. Many studies indicate that song characteristics are not only related to individual fitness but also to male age [10], [11]. In several species, older males have bigger repertoires [12] or perform songs better than one year old males [13], [14]. Age and reproductive success are often interrelated, as age is an indicator of male viability [15]. Indeed, in several songbirds breeding success has been found to increase with age [16], [17]. Accordingly, males may signal their age and quality with their songs [14], [18], [19], [20], [21].

In the context of male quality signalling, one song parameter that has been neglected by previous studies is song amplitude. This is surprising, for the potential of male song amplitude for sexual selection was highlighted by Gil and Gahr [1] a decade ago. In recent years, methods have been developed for reliably measuring vocal amplitude in free-ranging birds [22], [23], [24], [25], [26]. Song amplitude is a flexible trait that can be individually regulated, for instance in relation to environmental noise [27], [28] or social context [23], [29], [30], [31]. Increasing the amplitude of a song is a successful tactic to communicate over long distances and in high noise levels [32], [33]. However, in addition to individual adjustments in song amplitude, several studies have found consistent differences in vocal amplitude between males of a population [26], [34], [35], [36]. In insects and anurans, variation in male vocal amplitude plays an important role in both female choice and male-male competition [37]. Several studies indicate that this is also true in birds: females of several species have been found to prefer louder songs [38], [39] and song amplitude is also important in male-male territorial interactions [23], [26], [34]. Thus, the sound pressure level is likely to have a function in both inter- and intra-sexual selection. However, it is unknown whether and how the striking variation in song amplitude between males varies with fitness.

We addressed this topic in a field study on rock sparrows (*Petronia petronia*). The overall aim of the study was to investigate whether male rock sparrows use their advertisement songs to signal their age and fitness. In particular, we related song parameters, including song amplitude, to the singer's age and reproductive success of the current mating season. The main reason to choose the rock sparrow as study species is a technical one: rock sparrows are not territorial but breed semi-colonially, i.e. they defend only one or two meters around their nest against other males [40]. In our study population, males mostly sing on top of their nestboxes. This enabled us not only to predict the males' song posts but also to place a radio microphone in a fixed position above the singing male, which is the ideal position to reliably measure the sound pressure level of song. The breeding biology of the species has been intensively studied regarding plumage ornaments that are present in both sexes [41], [42], [43], [44], but much less is known about the role of male songs in sexual selection. Rock sparrows are mainly socially monogamous, but some males are socially polygynous, and a considerable proportion of young is sired through extra-pair matings [45]. Therefore we assessed fitness as the genetic breeding success of males based on paternity analysis with microsatellite markers.

## Methods

### Ethics statement

The protocols for capturing, handling, marking, observing and taking blood were approved by the ethical committee of the Max Planck Institute for Ornithology and the Natural History Museum Paris. E.N. holds a permanent German ringer license (Radolfzell ringer No. 1694) and had a temporary French ringer license for 2008 and 2009 approved by the French Centre de Recherches par le Baguage de Populations d'Oiseaux.

### Study species and study site

Rock sparrows were studied in the Clarée valley in the French Alps close to the villages of Les Alberts and Nevache. The local population has been studied as part of a long-term project by G. M. since 2001 [40], [46], [47]. The birds of this population bred almost exclusively in wooden nestboxes (10×34 cm and 10 cm high) that were attached to utility poles at a height of four meters. The distance between neighbouring nest boxes was approximately 50 m. Since the population has been colour-ringed and monitored for several years, we were able to determine the exact age of many individuals. Unknown birds that may have immigrated from other localities were caught and colour-ringed at the beginning of each breeding season. Birds were caught by nestbox-traps or mistnets. All data reported here were gathered in 2008 and 2009. Our study population comprised 31 territorial males in 2008 and 33 males in 2009. We could get paternity data for 18 territorial males in 2008, and in 2009 we gathered genetic data from 31 males. Five of these males bred in both study years, and we used their genetic and acoustic data from only one year (decided by coin toss). We tried to get song recordings for all males in the early phase of the breeding season before the onset of egg laying in the own nest. During this phase, males sing often on top of their nest box [40], and they are visited by different females, who investigate the nestbox and may or may not become the future partner of the resident male. Even during the nest building phase more than one female usually visits the nest. Therefore we decided to use the whole period of the early breeding season until egg-laying as our time unit for analysis. Our sample size was limited by the necessity to get recordings of males singing on the nestbox under the microphone to measure the sound pressure level of their song. In total, we could record 25 different males that could be used for the analyses of song variables in relation to breeding success. Twenty-two of these 25 males bred successfully, and the song data from these males were used to analyse the relation between paternity loss and song parameters. Three additional males occupied nest boxes but remained unmated. Their songs were recorded before the last female in the population started to lay eggs. The songs of an additional three unringed, but mated males were used in the calculation of the variation of song amplitude within males. The relation between overall reproductive success and song parameters were both analysed with the 22 paired males and the extended dataset of 25 males. We were able to determine the age of 22 males in our sample based on ringing data from previous years and for a subset of 20 of those males we also measured reproductive success. The onset of egg-laying did not differ between females of yearlings and females of older males (Mann-Whitney U-test, U = 34.5. N_older_ = 12, N_yearling_ = 7, p = 0.54).

### The song of rock sparrows

**Figure 1:**
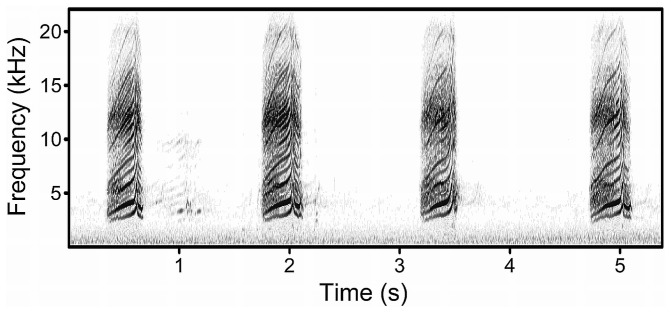
An example of a typical rock sparrow advertisement song. A song bout consists of one element that is repeated several times. Song performance (element rate and song amplitude) may vary considerably between males.

The advertisement song of the species consists of only one element that is repeated several times forming a song bout (Fig. 1). Similar to strophes in other bird species, rock sparrow song elements are separated by pauses of 2.0 to 4.7 seconds and therefore we use hereafter the term element and song synonymously. Males sing in the immediate vicinity to the nest, often with no female nearby. Another, much rarer, type of song that we did not consider here is the courtship song. Courtship song is structurally different from the advertisement song and is produced only in the presence of females, often immediately before a copulation [47]. The male advertisement song is primarily used in male-female interactions [41], [47]. In particular, territorial males sing at a higher rate when a female is approaching the nesting site (Matessi, unpublished data). However, it remains to be shown whether the intra-sexual function of song is mainly related to the attraction of females or to strengthen the pair bond.

### Microsatellite analysis

To determine parentage we sampled 5–25 µl blood from the brachial vein of adults and 13 days old chicks. In total, we took blood samples from 44 adult males (76% of the reproducing males), 42 adult females (90% of the reproducing females) and 285 young (100% of young). The blood was immediately suspended and stored in Queen's lysis buffer [48]. DNA was isolated with the GFX Genomic Blood DNA Purification Kit (GE Healthcare Europe, Freiburg, Germany). For paternity analysis, we used eleven microsatellite primers developed for this species by Grapputo et al. [49]. Microsatellite amplifications were performed in multiplexed PCRs using the QIAGEN Multiplex PCR kit (QIAGEN) and primer mixes containing three to four primer pairs, using a GeneAmp PCR System 2007 (Applied Biosystems). Forward primers were labeled at their 5′ end with fluorescent dyes from Applied Biosystems. Differences in amplification efficiency and dye strength of the primers were accommodated by adapting the primer concentrations in these mixes. Concentrations and fluorescent dye of each forward and reverse primer in the primer mixes were as follows. Mix 1: 0.66 µM of PP1813 (PET), 0.33 µM of PP18117 (VIC), 1.33 µM of PP18113 (6FAM), 0.66 µM of PP18112 (6FAM); Mix 2: 0.66 µM of PP14 (VIC), 1 µM of PP15 (6FAM), 0.66 µM o1-13f PP18114 (PET); Mix 3: 0.5 µM of PP38 (6FAM), 0.5 µM of PP01 (VIC), 0.66 µM of PP18111 (NED). Each 10 µL multiplex PCR contained 20–60 ng DNA and was set up and amplified according to manufacturers instructions. Annealing temperature was 51°C for Mix 1 and 55°C for Mix 2 and 3. Microsatellite P11 and the sexing primers P2 and P8 (Griffiths et al. 1998) were amplified in single PCRs with standard protocols using 20–60 ng DNA, 0.25 U of Taq DNA polymerase (Fermentas), a provided buffer containing (NH_4_)_2_SO_4_ and 2 mM MgCl_2_. Annealing temperatures were 54°C for PP11 and 50°C for P2P8. 1.5 µl of the PCR product was mixed with formamide containing the GeneScan 500 LIZ Size Standard, heat denatured and resolved in POP4 polymer on an ABI 3100 Genetic Analyser (all Applied Biosystems). Raw data were analyzed with GeneMapper 4.0.

The 11 microsatellite markers showed 3–17 alleles in the study population (mean = 8). None of the markers deviated significantly from Hardy-Weinberg equilibrium. The combined probability of exclusion for the marker set was greater than 0.999. The combined non-exclusion probability for a pair of parents was 2.7×10^−7^. Paternity was excluded if loci showed multiple mismatches between the putative father and the offspring. In 199 cases there was no mismatch, in 10 cases one mismatch with the social father. We concluded that they were within-pair offspring and that the mismatches were due to mutations. Sixty-eight offspring showed 3–7 mismatches with the social father and were considered extra-pair young. For 40 offspring, another male in the population matched the paternal genotype completely (no mismatches). For two extra-pair offspring from different broods, another male had only one mismatch with the offspring. In one of these cases, the identified extra-pair male (zero mismatches) fathered another extra-pair offspring in the same brood.

In 2008 and 2009, we determined paternity for 277 of 285 young (97%), identified 30 different fathers and 40 different mothers and had 8 chicks with unknown father. We found a similar rate and distribution of extra-pair paternity in the two successive years: 22% (30/137) of the young were sired by an extra-pair father in 2008 and 27% (38/140) in 2009. We detected extra-pair young in 41% of all nests with identified paternity (12/29) in 2008 and in 48% (13/27) in 2009. For further analysis data from the two years were pooled.

### Song recordings

All birds were recorded between 0600 to 1054 hours during the early breeding season until the onset of egg-laying between 3 May and 27 June. Rock sparrows typically start singing in full daylight, i.e. there is no dawn chorus, and none of the measured song variables (see below) varied significantly with the time of day of the recording (Spearman rank-correlations: −0.29<r<0.19, N = 25, 0.16<P<0.96). Also, none of the measured song parameters correlated significantly with the recording date (−0.31<r<0.23, N = 25, 0.11<P<0.89). Song recordings were made with a wireless radio microphone system: a Sennheiser M2 omnidirectional Lavalier microphone was fixed to the utility pole facing downwards 65 cm above the nestbox. The microphone was connected to a Sennheiser SKP Evolution transmitter which was attached to the utility pole, out of sight of the bird. An observer at about 30 to 100 m distance received the signal with a Sennheiser EK 100 receiver and recorded it on one of two stereo tracks of a digital recorder (Marantz PMD 660 or Sound Devices 722 Digital Audio Recorder). During recording sessions, birds were observed with a scope and male behaviour was recorded on the second stereo track of the digital recorder. After each recording, we placed a loudspeaker (FoxPro Scorpion model X1-A) at the position of the singing male to calibrate the recording; a reference signal (4 kHz sine tone) with known amplitude was broadcasted and recorded with the same settings as the bird song. The amplitude of the reference signal was measured in an anechoic chamber with a CEL-573 Class 1 precision sound level analyzer. The reference signal was used to calibrate Avisoft SASLab Pro (version 5.0.13) software for the subsequent sound pressure level measurements. All sound pressure measurements are given as SPL values, i.e. in relation to 20 µPa. For the song analyses (see below) we used only recordings of song bouts with at least 8 songs. In total 1248 songs were used in the analysis (range 8 to 309 songs per male). Fifteen males were recorded in 2008 and 13 different males in 2009. There was no significant difference between the two cohorts from the two successive years in any of the measured frequency and temporal variables (see below), nor in amplitude (Mann-Whitney U-test: 70<U<94, N_1_ = 15, N_2_ = 13, 0.22<P<0.89). Hence, we pooled the data of both years for further analyses.

### Song analyses

Song recording files were copied to a computer and then analysed with the software Avisoft SASLab Pro (version 5.0.13). Spectral parameters were measured with a frequency resolution of 86 Hz and temporal variables with a resolution of 5.8 ms. Because our radio microphone system allowed us to record the songs with high signal-to-noise ratios, the acoustic analyses could be done using the automatic parameter measurement function of Avisoft. For each element of a song bout, we measured the following parameters: peak frequency (i.e. the frequency at the maximum amplitude in the spectrum), minimum and maximum frequency (i.e. the lowest and highest frequency 20 dB below the peak amplitude), and sound pressure level. From these values, averages were calculated for each individual, which were then used for further analysis. In addition, we measured the duration of all elements and the singing rate (number of song elements per minute) for each song bout. Again, average values were calculated in cases where more than one song bout was recorded for each individual. The distribution of all measured song variables did not deviate from normality (Kolmogorov-Smirnov test, N = 28, 0.47<P<0.86). Sets of raw variables as in our study are often reduced with a principal component analysis to yield a lower number of composite measures. However, our raw variables showed too little covariation and the Kaiser-Meyer-Olkin measure of sampling adequacy indicated that our data set was not suited for a principal component analysis (KMO measure <0.5, [50]). Therefore, we analyzed each raw variable separately.

**Figure 2:**
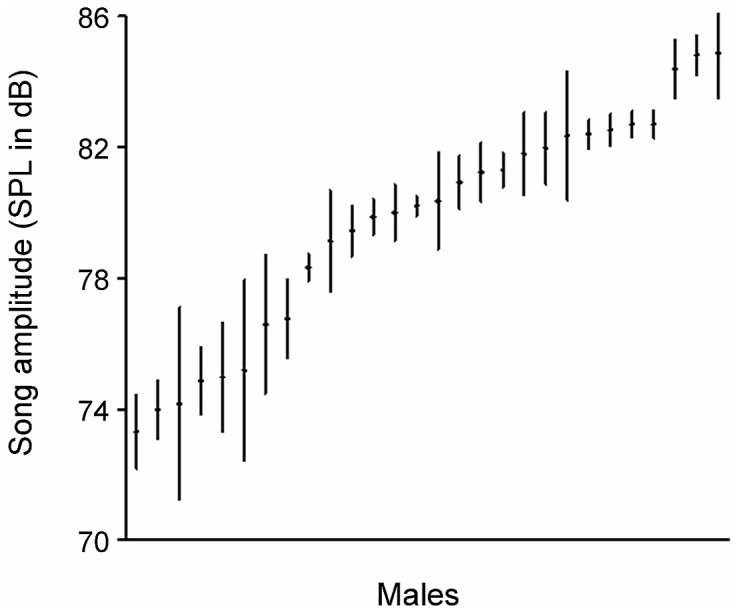
Variation of song amplitude within and between male rock sparrows. Values are shown as means ±95% confidence intervals.

The average song amplitude in our sample of 28 males ranged from 73 to 85 dB SPL. This 12 dB maximum difference between males was bigger than the variation within males (Fig. 2). To further assess the variation of acoustic measurements within males, we calculated a repeatability score from a one-way ANOVA with individual as factor [51]. In this analysis, all males were included for which we had two or more recordings of different song bouts (N = 14 males). Between these song bouts males left their song post for 8 to 98 minutes. These males showed a high repeatability of song amplitude measurements, and medium to high levels of repeatability in frequency and temporal measurements (based on one-way ANOVAs with DF_between_ = 13 and DF_within_ = 16): amplitude, repeatability R = 0.701±0.11 SE (F = 5.70, P = 0.0007), peak frequency, R = 0.42±0.12 SE, (F = 2.44, P = 0.04), mimimum frequency, R = 0.56±0.12 SE (F = 3.50, P = 0.009), maximum frequency R = 0.69 (F = 5.50, P = 0.09), song duration, R = 0.63±0.12 SE (F = 4.39, P = 0.004) and song rate, R = 0.93±0.032 SE (F = 27.8, P<0.001).

### Analysis of paternity, breeding success and age

We constructed generalized linear models (GLMs) to analyse song variables in relation to reproductive success and age. Full models were simplified by excluding variables in order of decreasing significance until only terms with P<0.1 remained in the model. Stepwise methods might produce inflated Type I errors [52] and therefore we present both full and reduced models. To avoid multi-collinearity [53] we excluded variables among our predictor variables that were highly correlated (r>0.4, Table 1). Thus, for each of the four dependent variables (i.e. overall breeding success, paternity gain, paternity loss, and age) we initially ran four models and for each model we calculated a full model and then a reduced model that retained only the significant predictor variables. Maximum frequency correlated with peak frequency and song rate varied with song duration (Table 1). In all final models, maximum frequency had a stronger effect on the dependent variables than peak frequency, and song rate had a greater explanatory power than song duration. Therefore, we report only the models with maximum frequency and song rate.

**Table 1:** Pearson correlation matrix of song parameters (N = 28 males).

	Song duration	Peak frequency	Minimum frequency	Maximum frequency	Amplitude
Song rate	**0.478** *	0.14	0.16	0.00	−0.05
Song duration		−0.08	−0.03	−0.13	0.27
Peak frequency			**0.76** **	**0.69** **	−0.01
Minimum frequency				0.27	0.16
Maximum frequency			.		−0.03

Significant correlations are indicated in bold.

* . Correlation is significant at the 0.05 level (2-tailed).

** . Correlation is significant at the 0.01 level (2-tailed).

Depending on the distribution of the dependent variables we used different types of GLMs. Paternity loss was a binary variable and we constructed the respective models with binomial error structure and logit-link function. For paternity gain (measured as the number of young sired in other nests) we used models with a quasi-Poisson error structure and a log-link function, because models with a Poisson error structure showed overdispersion [54]. For breeding success we constructed models with Gaussian error structure. Values of individual breeding success ranged from 0–10 young. Models were calculated for all paired males and also for the extended data set with three additional unpaired males. In both cases, the distribution of the values for breeding success did not deviate significantly from normality (paired males, mean ± SE breeding success, 5.5±0.50, Kolmogorov-Smirnov test, N = 22, Z = 0.887, P = 0.411; paired plus unpaired males, mean ± SE breeding success, 4.84±0.57, N = 25, Z = 0.724, P = 0.671). To investigate song as a potential indicator of age, we split our sample into two age classes: yearling males and males older than one year (older). Age was thus analysed as a binary variable by constructing GLMs with binomial error structure. All statistical analyses were performed with R.2.12.2.

## Results

### Age, breeding success and extra-pair paternity

Older males had a higher breeding success than yearlings (median and inter-quartile range (IQR), older males: 5.75, 5.75–7.00, yearlings: 5.00, 0.00–5.00, Mann-Whitney U-test: U = 17.5, N_yearling_ = 8, N_older_ = 12, P = 0.01). This was due to a significantly higher number of extra-pair young sired by older males (median and IQR, older males: 2.00, 0.50–3.00, yearlings: 0.00, 0.00–0.25, U = 16.0, N_yearling_ = 8, N_older_ = 12, P = 0.021). Despite their greater overall reproductive success, older males had more extra-pair young in their own nests, though this difference failed to reach significance (median and IQR, older males: 1.00, 0.00–2.50, yearlings: 0.00, 0.00–0.00, U = 27.0, N_yearling_ = 8, N_older_ = 12, P = 0.06). Two of the older males in our sample bred with two females, and these socially polygynous males had a relatively high breeding success. When we excluded the polygynous males from the analysis, we still found that older males had a higher breeding success (median and IQR, older males: 6.00, 5.25–6.00, yearlings: 5.00, 0.00–5.00, U = 16.5, N_yearling_ = 8, N_older_ = 10, P = 0.03), a higher paternity gain (median and IQR, older males: 1.00, 0.00–3.00, yearlings: 0.00, 0.00–0.25, U = 21.5, N_yearling_ = 8, N_older_ = 10, P = 0.03), and also lost almost significantly more paternity in their own nest (median and IQR, older males: 0, 0–1, Yearlings: 0, 0–0, U = 24.5, N_yearling_ = 8, N_older_ = 10, P = 0.06).

#### Song as an indicator of breeding success, extra-pair paternity and age

**Figure 3:**
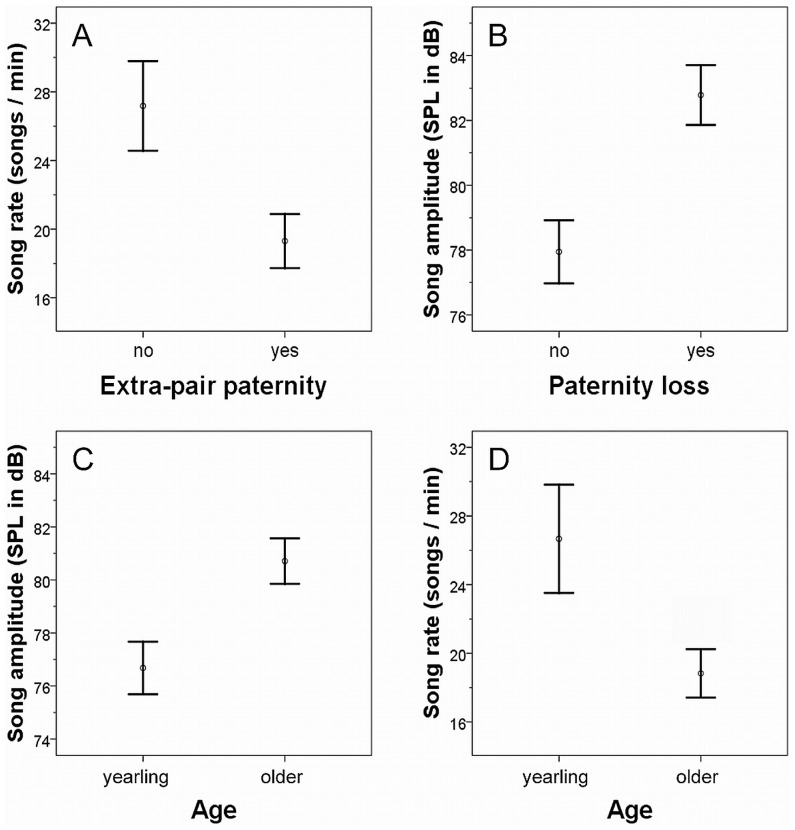
Differences in song performance in relation to paternity gain, paternity loss, and male age. All values are shown as means ± standard error. N_gain_ = 14, N_no gain_ = 11, N_loss_ = 11, N_no loss_ = 11, N_yearlings_ = 9, and N_older_ = 13.

The measured song variables explained some of the variation in breeding success, extrapair gain and paternity loss (Table 2, Fig. 3a, 3b). Males singing with high maximum frequencies and at low song rates sired more offspring (mean of maximum frequency, 5.7±0.21 kHz; Table 2). In the data set of 22 males (excluding unpaired males), these two song variables were also significant in the final model: (GLMs, DF = 20, estimate ± standard error, song rate: −0.10±0.044, t = −2.099. P = 0.049; maximum frequency: 1.33±0.41. t = 3.219, P = 0.005).

**Table 2:** Song variables that explained variation in breeding success, number of sired extra pair chicks and paternity loss in rock sparrows by GLMs.

Dependent variable: Breeding success (N = 25)				
Predictors	Estimate	SE	t	P-value
*Full model*				
Intercept	−2.63	10.48	−0.25	0.80
Amplitude	0.02	0.13	0.13	0.89
Song rate	−0.13	0.06	−2.37	0.027
Minimum frequency	0.41	1.02	0.40	0.69
Maximum frequency	1.43	0.51	2.79	0.011
*Reduced Model*				
Intercept	−0.55	2.95	−0.19	0.85
Song rate	−0.13	0.05	−2.44	0.023
Maximum frequency	1.49	0.47	3.19	0.004

Dependent Variable: Extra-pair gain (N = 25)				
Predictors	Estimate	SE	t	P-value
*Full model*				
Intercept	1.10	6.32	0.17	0.86
Amplitude	−0.07	0.08	−0.89	0.38
Song rate	−0.20	0.09	−2.22	0.04
Minimum frequency	1.24	0.83	1.50	0.15
Maximum frequency	0.91	0.50	1.82	0.087
*Reduced Model*				
Song rate	−0.12	0.05	−2.42	0.025
Maximum frequency	0.48	0.16	2.94	0.008

Dependent variable: Paternity loss (N_yearling_ = 11, N_adult_ = 11)				
Predictors	Estimate	SE	z	P value
*Full model*				
Intercept	−50.20	20.62	−1.99	0.047
Amplitude	0.63	0.3	1.99	0.046
Song rate	−0.02	0.08	−0.25	0.78
Minimum frequency	1.26	1.76	0.72	0.47
Maximum frequency	−0.36	0.69	−0.52	0.60
*Reduced model*				
Intercept	−48.40	22.14	−2.19	0.028
Amplitude	0.60	0.27	2.19	0.028

Similar to overall breeding success, low song rates also indicated extra-pair success, because males that gained extra-pair paternities produced fewer song elements per minute (Fig. 3a). In addition, males that gained extra-pair paternities also sang at a higher pitch (Table 2). Males that lost paternity in their nests produced songs with significantly higher amplitudes (Fig. 3b, Table 2). On average, cuckolded males sang 4.8 dB louder than those that did not loose paternity in their own nest (mean ± SE, amplitude cuckolded males, 82.8±0.92 dB, non-cuckolded males: 77.9±0.92 dB).

We found no significant differences when we analysed the pair-wise differences of song variables between social mates and extra-pair mates (exact Wilcoxon signed rank tests, 22<W<33.5, N = 7, 0.156<P<0.938). However, the lack of significance can be due to the small sample size.

Song performance characteristics varied between the two age classes with older males singing at a lower rate and at higher amplitudes than yearlings (Fig. 3c and 3d). However, song rate as predictor variable was significant only in the final model and song amplitude marginally failed to reach statistical significance (Table 3).

**Table 3:** Song variables that predicted the two age classes by GLMs (age as binary dependent variable, yearlings vs. older males).

Dependent variable: Age (N_yearling_ = 8, N_older_ = 13)				
Predictors	Estimate	SE	z	P-value
*Full model*				
Intercept	27.01	19.13	−1.41	0.16
Amplitude	0.36	0.27	1. 344	0.18
Song rate	−0.31	0.16	−1.85	0.064
Minimum frequency	1.24	2.32	0.54	0.59
Maximum frequency	0.43	0.74	0.59	0.56
*Reduced Model*				
Intercept	−28.67	18.26	−1.57	0.12
Amplitude	0.45	0.25	1.80	0.072
Song rate	−0.26	0.13	−2.07	0.038

## Discussion

Our results indicate that male rock sparrow songs reflect age and reproductive success of the singer. Males with high reproductive success sang with higher maximum frequencies and at low song rates, whereas paternity loss was reflected in high song amplitude. At the same time, song amplitude and song rate also varied with male age: the two age classes could be predicted by a lower song rate and higher amplitudes in old males.

In our study population, we observed an extra-pair paternity rate of 25% extra-pair young, which is high in comparison to other songbirds [55] and similar to the rate found in another rock sparrow population [45]. In this other population, Pilastro et al. [45] found a higher rate of cuckoldry in polygynous as well as in older males. They could also show that males with more intense mate-guarding behaviour suffered less paternity loss and they concluded that there is a trade-off between mate guarding and attracting a new mate in polygynous individuals. Our data confirm a higher rate of cuckoldry in older males but this pattern was even found when we excluded polygynous males from the analysis. In contrast to the study of Pilastro et al. [45] we used microsatellite analysis instead of DNA fingerprinting and were thus able to identify extra-pair fathers. We found that older males surpassed the paternity loss in their own nest by gaining more extra-pair paternity in other nests. So, overall reproductive success was significantly higher in older males than in yearlings. This result is consistent with other studies that show a greater reproductive performance in older birds [16], [56], [57].

Interestingly, female rock sparrows may assess the quality of a potential mate by his song. We found that older males differed in their song performance from younger males that had a lower reproductive success. The acoustic characteristic that signalled both age and reproductive success was the song rate; more successful and older males sang with lower song rate. More successful males sang also with significantly higher maximum frequency, a feature that did not differ between yearlings and older males. Our findings on song rate are in contrast to several studies that have suggested a positive relationship between song rate and male quality [58], [59], [60]; but see Forstmeier [61]. However, Garamszegi et al. [62] found that in collared flycatchers (*Ficedula albicollis*), yearlings also sing faster than older males, and the authors offered two explanations for their unexpected result. First, they argued that the higher song rate of yearlings could be a compensation for the higher repertoire size in older collared flycatchers. Second, higher song rate could be a consequence of higher aggression in yearlings and song rate could be rather related to more intense territorial defence. The latter might also explain our findings in rock sparrows. Young males might mitigate their inferior quality with higher aggression levels, which would be in turn reflected in higher song rates. Higher aggression levels may also account for the lower song pitch of less successful males in our study. This notion is supported by findings from anurans, in which males may lower the dominant frequency of their calls during disputes, and the magnitude of this frequency shift is associated with a greater probability of attacking the rival male [63], [64]. In birds some studies support a negative correlation between aggression and frequency [65], [66], while others find the opposite relationship [67], [68].

However, whether female birds actually use song rate or pitch in their mating decisions remains an open question. A clear relationship of how male signals relate to age and female preference was demonstrated in the field cricket *Gryllus campestris*. In this species, the carrier frequency is the main song component under female preference [69] and as males get older, their song frequency decreases [70]. Thus, by choosing males singing at a low frequency, female crickets base their mate choice decision on a sexual signal that indicates viability. Other studies on closely related species also found predictable changes of male call characteristics with age [71], [72], [73]. However, Judge [72] suggests that the preference of female *Gryllus pennsylvanicus* for older males may actually be a side-effect of discrimination against heterospecific matings. Moreover, *Gryllus bimaculatus* females showed the opposite pattern and preferred the songs of younger males [73]. Similarly, our results on song rate do not conform to the prevailing generalization about age signalling in bird song [11], since high quality males produced less intense signals.

Another performance-related signal trait, song amplitude, was successfully measured with our new radio microphone method, which allowed a more straightforward and economical recording of vocal amplitudes than previous methods [22], [24], [26]. However, our assay is limited to circumstances in which the song perch of a male can be predicted, as was the case in our nestbox population. We found a consistent 12 dB maximum difference in song amplitude between males, which is similar to findings in other species (14 dB in nightingales *Luscinia megarhynchos* [22], 9 dB in chaffinches *Fringilla coelebs* [26]). The observed individual variation in song amplitude did not predict variation in paternity gain or in overall breeding success. Song amplitude failed marginally to reach statistical significance, although older males had (1) a higher overall reproductive success and (2) they produced louder songs than yearlings. This is surprising because in other bird species females have been found to prefer louder songs [38], [39]. In insects and anurans, females prefer louder mating signals because signal amplitude reflects male size [37]. In songbirds, however, song amplitude does not vary with body size [35]. Most remarkably, we found a negative relationship between song amplitude and paternity loss: males that lost paternity in their own nest sang significantly louder than those that did not. Older males, which generally had a higher overall reproductive success, also sang louder, but older males also lost more paternity.

Perhaps, the louder songs in rock sparrows are not the cause of paternity loss, but a consequence: males may vary their song amplitude in relation to the strength of the pair bond with their social females, so that they would e.g. sing louder if their mate is absent more often or if their mate is further away. Thereby, males could increase the active space of their songs and thus keep in contact with their mate [29]. A similar negative relationship between vocal amplitude and male quality was shown in bisons (*Bison bison*), in which males with lower reproductive success produced louder rutting calls [74]. We also note that in many songbirds, males that lost their mate start singing loudly throughout the day, and temporary mate removal typically causes increased singing [75]. By and large, our findings show that male advertisement songs in rock sparrows reflect important information that may be used in intersexual selection. Thus, our study supports the notion that male songs are primarily directed at females in this species. However, territorial males react aggressively towards rival males close to their nests (Matessi unpublished data) and an increased song rate may also be a component of this territorial behaviour [40].

We have to bear in mind, that the reported relationships between song amplitude and fitness in rock sparrows are based on correlational data. As a next step we would need experimental evidence to confirm the function of song amplitude in this species. In other songbirds, loud songs are used as a signal of territorial threat [26], [76] or as an indicator of high current condition [77]. Our study suggests a more complex picture in rock sparrows, indicating that vocal amplitude may have different functions in different species.

## References

[1] Gil D, Gahr M (2002) The honesty of bird song: multiple constraints for multiple traits. Trends Ecol Evol 17: 133–141.

[2] Catchpole CK, Dittami J, Leisler B (1984) Differential responses to male song repertoires in female songbirds implanted with oestradiol. Nature 312: 563–564.

[3] Searcy WA, Marler P (1984) Interspecific differences in the response of female birds to song repertoires. Z Tierpsychol 66: 128–142.

[4] Soma M, Garamszegi LZ (2011) Rethinking birdsong evolution: meta-analysis of the relationship between song complexity and reproductive success. Behav Ecol 22: 363–371.

[5] Vallet E, Beme I, Kreutzer M (1998) Two-note syllables in canary songs elicit high levels of sexual display. Anim Behav 55: 291–297. 9480696 10.1006/anbe.1997.0631

[6] Ballentine B, Hyman J, Nowicki S (2004) Vocal performance influences female response to male bird song: an experimental test. Behav Ecol 15: 163–168.

[7] Byers BE (2007) Extrapair paternity in chestnut sided warblers is correlated with consistent vocal performance. Behav Ecol 18: 130–136.

[8] Poesel A, Kunc HP, Foerster K, Johnsen A, Kempenaers B (2006) Early birds are sexy: male age, dawn song and extrapair paternity in blue tits *Cyanistes* (formerly *Parus*) *caeruleus* . Anim Behav 72: 531–538.

[9] Suter SM, Ermacora D, Rieille N, Meyer DR (2009) A distinct reed bunting dawn song and its relation to extrapair paternity. Anim Behav 77: 473–480.

[10] Garamszegi LZ, Heylen D, Moller AP, Eens M, de Lope F (2005) Age-dependent health status and song characteristics in the barn swallow. Behav Ecol 16: 580–591.

[11] Kipper S, Kiefer S (2010) Age-related changes in birds' singing styles: on fresh tunes and fading voices? Adv Study Behav 41: 77–118.

[12] Kiefer S, Spiess A, Kipper S, Mundry R, Sommer C, et al (2006) First-year common nightingales (*Luscinia megarhynchos*) have smaller song-type repertoire sizes than older males. Ethology 112: 1217–1224.

[13] Ballentine B (2009) The ability to perform physically challenging songs predicts age and size in male swamp sparrows, *Melospiza georgiana* . Anim Behav 77: 973–978.

[14] de Kort SR, Eldermire ERB, Valderrama S, Botero CA, Vehrencamp SL (2009) Trill consistency is an age-related assessment signal in banded wrens. Proc R Soc Lond B Biol Sci 276: 2315–2321. 10.1098/rspb.2009.0127 PMC2677607 19324742

[15] Andersson M (1994) Sexual selection. Princeton, NJ: Princeton University Press.

[16] Kempenaers B, Verheyren GR, Dhondt AA (1997) Extrapair paternity in the blue tit (*Parus caeruleus*): female choice, male characteristics, and offspring quality. Behav Ecol 8: 481–492.

[17] Geslin T, Questiau S, Eybert MC (2004) Age-related improvement of reproductive success in Bluethroats *Luscinia svecica* . Bird Study 51: 178–184.

[18] Radesäter T, Jakobsson S, Andbjer N, Bylin A, Nyström K (1987) Song rate and pair formation in the willow warbler, *Phylloscopus trochilus* . Anim Behav 35: 1645–1651.

[19] Botero CA, Rossman RJ, Caro LM, Stenzler LM, Lovette IJ, et al (2009) Syllable type consistency is related to age, social status and reproductive success in the tropical mockingbird. Anim Behav 77: 701–706. 20161558 10.1016/j.anbehav.2008.11.020 PMC2805192

[20] de Kort SR, Eldermire ERB, Cramer ERA, Vehrencamp SL (2009) The deterrent effect of bird song in territory defense. Behav Ecol 20: 200–206. 19337589 10.1093/beheco/arn135 PMC2662740

[21] Rivera-Gutierrez HF, Pinxten R, Eens M (2010) Multiple signals for multiple messages: great tit, *Parus major*, song signals age and survival. Anim Behav 80: 451–459.

[22] Brumm H (2004) The impact of environmental noise on song amplitude in a territorial bird. J Anim Ecol 73: 434–440.

[23] Brumm H, Todt D (2004) Male-male vocal interactions and the adjustment of song amplitude in a territorial bird. Anim Behav 67: 281–286.

[24] Nemeth E (2004) Measuring the sound pressure level of the song of the screaming piha *Lipaugus vociferans*: one of the loudest birds in the world? Bioacoustics 14: 225–228.

[25] Patricelli GL, Dantzker MS, Bradbury JW (2008) Acoustic directionality of red-winged blackbird (*Agelaius phoeniceus*) song relates to amplitude and singing behaviours. Anim Behav 76: 1389–1401.

[26] Brumm H, Ritschard M (2011) Song amplitude affects territorial aggression of male receivers in chaffinches. Behav Ecol 22: 310–316.

[27] Cynx J, Lewis R, Tavel B, Tse H (1998) Amplitude regulation of vocalizations in noise by a songbird, *Taeniopygia guttata* . Anim Behav 56: 107–113. 9710467 10.1006/anbe.1998.0746

[28] Brumm H, Todt D (2002) Noise-dependent song amplitude regulation in a territorial songbird. Anim Behav 63: 891–897.

[29] Brumm H, Slater PJB (2006) Animals can vary signal amplitude with receiver distance: evidence from zebra finch song. Anim Behav 71: 699–705.

[30] Dabelsteen T, McGregor P, Lampe HM, Langmore N, Holland J (1998) Quiet song in song birds: an overlooked phenomenon. Bioacoustics 9: 89–105.

[31] Anderson RC, Nowicki S, Searcy WA (2007) Soft song in song sparrows: response of males and females to an enigmatic signal. Behav Ecol Sociobiol 61: 1267–1274.

[32] Brumm H, Naguib M (2009) Environmental acoustics and the evolution of bird song. Adv Study Behav 40: 1–33.

[33] Brumm H, Robertson KA, Nemeth E (2011) Singing direction as a tool to investigate the function of birdsong: an experiment on sedge warblers. Anim Behav 81: 653–659.

[34] Dabelsteen T (1981) The sound pressure level in the dawn song of the blackbird *Turdus merula* and a method for adjusting the level in experimental song to the level in natural song. Z Tierpsychol 56: 137–149.

[35] Brumm H (2009) Song amplitude and body size in birds. Behav Ecol Sociobiol 63: 1157–1165. 10.1007/s00265-009-0749-y PMC2699386 19554102

[36] Ritschard M, Brumm H (2011) Effects of vocal learning, phonetics and inheritance on song amplitude in zebra finches. Anim Behav 82: 1415–1422.

[37] Gerhardt H, Huber F (2002) Acoustic communication in insects and anurans. Chicago; The University of Chicago Press. 542p.

[38] Searcy WA (1996) Sound pressure levels and song preferences in female red-winged blackbirds (*Agelaius phoeniceus*) (Aves, Emberizidae). Ethology 102: 187–196.

[39] Ritschard M, Riebel K, Brumm H (2010) Female zebra finches prefer high-amplitude song. Anim Behav 79: 877–883.

[40] Matessi G, McGregor PK, Peake TM, Dabelsteen T (2005) Do male birds intercept and use rival courtship calls to adjust paternity protection behaviours? Behaviour 142: 507–524.

[41] Pilastro A, Griggio M, Matessi G (2003) Male rock sparrows adjust their breeding strategy according to female ornamentation: parental or mating investment? Anim Behav 66: 265–271.

[42] Griggio M, Valera F, Casas A, Pilastro A (2005) Males prefer ornamented females: a field experiment of male choice in the rock sparrow. Anim Behav 69: 1243–1250.

[43] Griggio M, Morosinotto C, Pilastro A (2009) Nestlings' carotenoid feather ornament affects parental allocation strategy and reduces maternal survival. J Evol Biol 22: 2077–2085. 19694895 10.1111/j.1420-9101.2009.01823.x

[44] Griggio M, Serra L, Licheri D, Monti A, Pilastro A (2007) Armaments and ornaments in the rock sparrow: a possible dual utility of a carotenoid-based feather signal. Behav Ecol Sociobiol 61: 423–433.

[45] Pilastro A, Griggio M, Biddau L, Mingozzi T (2002) Extrapair paternity as a cost of polygyny in the rock sparrow: behavioural and genetic evidence of the ‘trade-off’ hypothesis. Anim Behav 63: 967–974.

[46] Matessi G, Carmagnani C, Griggio M, Pilastro A (2009) Male rock sparrows differentially allocate nest defence but not food provisioning to offspring. Behaviour 146: 209–223.

[47] Matessi G, McGregor PK, Peake TM, Dabelsteen T (2007) Female rock sparrows (*Petronia petronia*), not males, respond differently to simulations of different courtship interaction outcomes. Behaviour 144: 735–752.

[48] Seutin G, White BN, Boag PT (1991) Preservation of avian blood and tissue samples for DNA analysis. Can J Zool 69: 82–90.

[49] Grapputo A, Barbisan F, De Girolamo M, Pilastro A, Zane L (2006) Development and characterization of 11 microsatellite markers in the rock sparrow, *Petronia petronia* . Mol Ecol Notes 6: 1070–1072.

[50] Budaev SV (2010) Using principal components and factor analysis in animal behaviour research: caveats and guidelines. Ethology 116: 472–480.

[51] Lessells CM, Boag PT (1987) Unrepeatable repeatabilities. Auk 104: 116–121.

[52] Mundry R, Nunn CL (2009) Stepwise model fitting and statistical inference: turning noise into signal pollution. Am Nat 173: 119–123. 19049440 10.1086/593303

[53] Tabachnick BG, Fidell LS (2007) Using Multivariate Statistics. 5th edition, Boston: Allyn and Bacon. 980p.

[54] Crawley MN (2007) The R Book. Chichester, UK: John Wiley & Sons, Ltd. 942p.

[55] Westneat DF, Stewart IRK (2003) Extra-pair paternity in birds: Causes, correlates, and conflict. Annu Rev Ecol Evol Syst 34: 365–396.

[56] Bouwman KM, Van Dijk RE, Wijmenga JJ, Komdeur J (2007) Older male reed buntings are more successful at gaining extrapair fertilizations. Anim Behav 73: 15–27.

[57] Schmoll T, Mund V, Dietrich-Bischoff V, Winkel W, Lubjuhn T (2007) Male age predicts extrapair and total fertilization success in the socially monogamous coal tit. Behav Ecol 18: 1073–1081.

[58] Welling PP, Rytkönen SO, Koivula KT, Orell MI (1997) Song rate correlates with paternal care and survival in willow tits: Advertisement of male quality? Behaviour 134: 891–904.

[59] Hofstad E, Espmark Y, Moksnes A, Haugan T, Ingebrigtsen M (2002) The relationship between song performance and male quality in snow buntings (*Plectrophenax nivalis*). Can J Zool 80: 524–531.

[60] Murphy MT, Sexton K, Dolan AC, Redmond LJ (2008) Dawn song of the eastern kingbird: an honest signal of male quality? Anim Behav 75: 1075–1084.

[61] Forstmeier W, Leisler B (2004) Repertoire size, sexual selection, and offspring viability in the great reed warbler: changing patterns in space and time. Behav Ecol 15: 555–563.

[62] Garamszegi LZ, Török J, Hegyi G, Szöllösi E, Rosivall B, et al (2007) Age-dependent expression of song in the collared flycatcher, *Ficedula albicollis* . Ethology 113: 246–256.

[63] Wagner WE (1992) Deceptive or honest signaling of fighting ability - a test of alternative hypotheses for the function of changes in call dominant frequency in male cricket frogs. Anim Behav 44: 449–462.

[64] Burmeister SS, Ophir AG, Ryan MJ, Wilczynski W (2002) Information transfer during cricket frog contests. Anim Behav 64: 715–725.

[65] Price JJ, Earnshaw SM, Webster MS (2006) Montezuma oropendolas modify a component of song constrained by body size during vocal contests. Anim Behav 71: 799–807.

[66] Geberzahn N, Goymann W, Muck C, ten Cate C (2009) Females alter their song when challenged in a sex-role reversed bird species. Behav Ecol Sociobiol 64: 193–204. 19946649 10.1007/s00265-009-0836-0 PMC2779343

[67] Araya-Ajoy YM, Chaves-Campos J, Kalko EKV, DeWoody JA (2009) High-pitched notes during vocal contests signal genetic diversity in ocellated antbirds. PLoS ONE 4: e8137. 19956580 10.1371/journal.pone.0008137 PMC2779863

[68] Ripmeester EAP, de Vries AM, Slabbekoorn H (2007) Do blackbirds signal motivation to fight with their song? Ethology 113: 1021–1028.

[69] Scheuber H, Jacot A, Brinkhof MWG (2004) Female preference for multiple condition-dependent components of a sexually selected signal. Proc R Soc Lond B Biol Sci 271: 2453–2457. 10.1098/rspb.2004.2907 PMC1691884 15590595

[70] Jacot A, Scheuber H, Brinkhof MWG (2007) The effect of age on a sexually selected acoustic display. Ethology 113: 615–620.

[71] Fitzsimmons LP, Bertram SM (2011) The calling songs of male spring field crickets (*Gryllus veletis*) change as males age. Behaviour 148: 1045–1065.

[72] Judge KA (2011) Do male field crickets, *Gryllus pennsylvanicus*, signal their age? Anim Behav 81: 185–194.

[73] Verburgt L, Ferreira M, Ferguson JWH (2011) Male field cricket song reflects age, allowing females to prefer young males. Anim Behav 81: 19–29.

[74] Wyman MT, Mooring MS, McCowan B, Penedo MCT, Hart LA (2008) Amplitude of bison bellows reflects male quality, physical condition and motivation. Anim Behav 76: 1625–1639.

[75] Kempenaers B, Lanctot RB, Robertson RJ (1998) Certainty of paternity and paternal investment in eastern bluebirds and tree swallows. Anim Behav 55: 845–860. 9632472 10.1006/anbe.1997.0667

[76] Ritschard M, van Oers K, Naguib M, Brumm H (2012) Song amplitude of rival males modulates the territorial behaviour of great tits during the fertile period of their mates. Ethology 118: 197–202.

[77] Ritschard M, Brumm H (2012) Zebra finch song reflects current food availability. Evol Ecol 26: 801–812.

